# Misuse of tumor marker levels leads to an insufficient International Germ Cell Consensus Classification (IGCCCG) risk group assignment and impaired treatment

**DOI:** 10.1002/cam4.6304

**Published:** 2023-07-01

**Authors:** Matthäus Majewski, Pia Paffenholz, Christian Ruf, Yue Che, Christoph Seidel, Julia Heinzelbecker, Hans‐Ulrich Schmelz, Cord Matthies, Peter Albers, Carsten Bokemeyer, Axel Heidenreich, Martin Pichler, Tim Nestler, the GTCSG (German Testicular Cancer Study Group)

**Affiliations:** ^1^ Department of Urology Federal Armed Services Hospital Ulm Ulm Germany; ^2^ Department of Urology University Hospital of Cologne Cologne Germany; ^3^ Department of Urology University Hospital of Duesseldorf Duesseldorf Germany; ^4^ Department of Oncology, Hematology and Bone Marrow Transplantation with Division of Pneumology University Medical Center Hamburg‐Eppendorf, University Hospital of Hamburg‐Eppendorf Hamburg Germany; ^5^ Department of Urology and Pediatric Urology University Medical Centre Homburg, Saarland University Hospital of Homburg Homburg Germany; ^6^ Department of Urology Federal Armed Services Hospital Koblenz Koblenz Germany; ^7^ Department of Urology Federal Armed Services Hospital Hamburg Hamburg Germany; ^8^ Department of Urology Medical University Vienna Vienna Austria; ^9^ Division of Oncology Medical University of Graz Austria

**Keywords:** AFP, hCG, IGCCCG, LDH, metastasis, NSGCT, serum tumor marker, testicular germ cell tumor

## Abstract

**Background:**

Metastatic germ cell tumors of the testis (GCTs) are risk‐stratified according to the International Germ Cell Cancer Collaborative Group (IGCCCG) classification system. This risk classification is based on anatomical risk factors as well as tumor marker levels of AFP, HCG, and LDH assessed pre‐chemotherapy after orchiectomy treatment. An incorrect classification is possible when pre‐orchiectomy marker levels are used, possibly resulting in over‐ or undertreatment of patients. The aim was to investigate the potential frequency and clinical relevance of incorrect risk stratification using pre‐orchiectomy tumor marker levels.

**Methods:**

A multicenter registry analysis, including patients with metastasized nonseminomatous GCT (NSGCT), was conducted by investigators of the German Testicular Cancer Study Group (GTCSG). Based on the marker levels at different timepoints, IGCCCG risk groups were calculated. The agreement was tested using Cohen's kappa.

**Results:**

A total of 672 of 1910 (35%) patients were diagnosed with metastatic NSGCTs, and 523 (78%) had sufficient data for 224 follow‐up data points. By using pre‐orchiectomy tumor marker levels, 106 patients (20%) would have been incorrectly classified. Seventy‐two patients (14%) were classified into a higher risk category, and 34 patients (7%) were classified into a lower risk category. Cohen's kappa was 0.69 (*p* < 0.001), showing a strong agreement between the use of both marker timepoints. The treatment of misclassified patients would have resulted in an overtreatment of 72 patients or undertreatment of 34 patients.

**Conclusions:**

The use of pre‐orchiectomy tumor marker levels may lead to an incorrect risk classification and might subsequently lead to under‐ or overtreatment of patients.

## 1 INTRODUCTION

Approximately half of all patients with a germ cell tumor of the testis (GCT) exhibit metastasis at the time of diagnosis or will develop metastasis during follow‐up.^1^, ^2^ Metastasized GCT should be risk stratified according to the prognostic International Germ Cell Cancer Collaborative Group (IGCCCG) classification system to determine the extent of necessary chemotherapy to obtain the best curative chance.^3^, ^4^, ^5^ Based on histological subtype (seminoma vs. nonseminoma), primary tumor location (testis or mediastinal extragonadal), location of metastasis and serum tumor marker levels pre‐chemotherapy (alpha‐fetoprotein (AFP), human chorionic gonadotropin (hCG) and lactate dehydrogenase (LDH)), patients with metastatic germ cell tumors are grouped into the “good”, “intermediate” or “poor” prognosis group (Table 1).

**TABLE 1:** IGCCCG risk classification group.

			Therapy
Good‐prognosis group			3 × BEP
Seminoma		Non‐Seminoma	
*All of the following criteria*			
No non‐pulmonary viceral metastase			
AFP normal		AFP < 1000 ng/mL and	
Any hCG		hCG < 5000 IU/L and	
Any LDH		LDH <1.5 × upper normal limit	
Any primary site		Testis/Retro‐peritoneal primary	
Intermediate‐prognosis group			4 × BEP
*All of the following criteria*		Testis/Retro‐peritoneal primary	
Non‐pulmonary visceral metastase		No non‐pulmonary viceral metastase and of the following criteria	
AFP normal			
Any hCG		AFP 1000–10,000 ng/mL or	
Any LDH		hCG 5000–50,000 IU/L or	
Any primary site		LDH 1.5–10 × upper normal limit	
Poor‐prognosis group			4 × BEP/therapy escalation
Not applicable		*Any of the following criteria*	
		Mediastinal primary	
		Non‐pulmonary visceral metastase	
		AFP > 10,000 ng/mL or	
		hCG > 50,000 IU/L or	
		LDH > 10 × upper normal limit	

Abbreviations: AFP, alpha‐fetoprotein; hCG, human chorionic gonadotrophin; LDH, lactate dehydrogenase.

The prognosis group determines the number of chemotherapy cycles after orchiectomy, namely, 3 cycles of BEP for good prognosis and 4 cycles of BEP for intermediate or poor prognosis, and in selected poor prognosis patients, therapy intensification may be needed (Table 1).^3^, ^4^

Pre‐orchiectomy tumor markers are elevated in approximately 60% of all patients.^6^ However, for a correct IGCCCG classification, serum tumor marker levels immediately prior to chemotherapy are needed. In daily clinical practice, it is frequently observed that initial pre‐orchiectomy tumor markers are used, which can lead to incorrect classification and under‐ or overtreatment. Undertreatment might lead to an impaired oncological outcome, while overtreatment might lead to chemotherapy‐associated, aggravated short‐ and long‐term toxicity, such as cardiovascular, renal, pulmonary or neuro‐ and ototoxicity, and an increased risk for secondary malignancies.^7^, ^8^, ^9^, ^10^, ^11^

We regularly receive requests from the “German second‐opinion network for testicular cancer” in which the presented metastatic GCT patients were misclassified with respect to their IGCCCG risk category because tumor marker levels at the time of orchiectomy were used.^12^ Therefore, our aim was to investigate the clinical relevance of an incorrect risk stratification according to the IGCCCG system when using pre‐orchiectomy tumor marker levels. The research questions were as follows: How many patients would be over‐ or undertreated? How many patients were incorrectly classified overall? What are the risks of an inappropriate therapy?

## 2 MATERIALS AND METHODS

**FIGURE 1:**
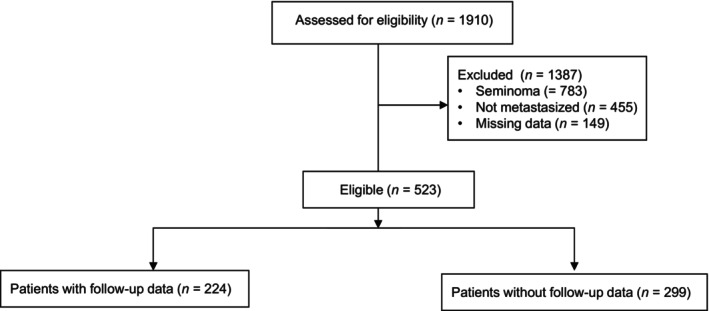
CONSORT diagram for patient selection.

This multicenter registry analysis was conducted by the German Testicular Cancer Study Group (GTCSG) in association with collaborators from Austria. Clinical information was collected retrospectively via pseudonymized electronic case report forms (eCRFs) from medical charts of the participating hospitals (Table 2). The eCRFs were subsequently centrally stored and assessed at the Department of Urology, Federal Armed Services Hospital Koblenz. The inclusion criteria for the main study were as follows: nonseminomatous GCT (NSGCT) of the testis and orchiectomy, status of metastasis, available serum marker levels prior to orchiectomy, and prior to chemotherapy. Patients who did not meet the inclusion criteria were excluded (Figure 1). Based on the pre‐orchiectomy and pre‐chemotherapy tumor marker levels, IGCCCG risk groups were calculated for each patient (Table 1).^3^ Next, we calculated whether the change in the risk group would have resulted in a different therapeutic regimen and checked which marker level was responsible for the deviating classification. For patients with follow‐up data, we reviewed whether the inaccurate classification resulted in altered survival.

**TABLE 2:** Participating hospitals.

Department of Urology, Federal Armed Services Hospital Ulm, Ulm, Germany
Department of Urology, University Hospital of Cologne, Cologne, Germany
Department of Urology, University Hospital of Duesseldorf, Duesseldorf, Germany
Department of University Hospital of Hamburg‐Eppendorf, Hamburg, Germany
Department of Urology and Pediatric Urology, University Medical Centre Homburg, Saarland University Hospital of Homburg, Homburg, Germany
Department of Urology, Federal Armed Services Hospital Koblenz, Koblenz, Germany
Department of Urology, Federal Armed Services Hospital Hamburg, Hamburg, Germany
Division of Oncology, Medical University of Graz, Graz, Austria

The study complied with the Declaration of Helsinki, and local ethics committee approval was inquired (University Hospital of Cologne 20‐1493). The Ethics Committee of the University Hospital of Cologne waived the need for ethics approval and the need to obtain consent for the collection, analysis and publication of the retrospectively obtained and anonymized data for this study.

### 2.1 Statistical analysis

Statistical analyses were performed using SPSS Statistics version 27.0 (IBM Corp.). The results for continuous normally distributed variables are expressed as the mean ± standard deviation (SD). Continuous nonnormally distributed variables are presented as the median and interquartile range (IQR), and categorical variables are presented as the number and percentage. All *p* values <0.05 were considered statistically significant.

To assess agreement between the IGCCCG risk group assignment based on pre‐chemotherapy versus pre‐orchiectomy marker levels, Cohen's kappa was used. According to Landis and Koch, a kappa of 0.61–0.8 was interpreted as substantial agreement, and that of 0.81–1.00 as excellent agreement.^13^

## 3 RESULTS

### 3.1 Patient characteristics

We collected data for 1910 patients, of whom 672 were patients with metastatic NSGCT. Of those, 523 (78%) were eligible according to the inclusion criteria. Reasons for ineligibility are listed in the CONSORT diagram (Figure 1). The patient and tumor characteristics of the NSGCT cohort are displayed in Table 3. The stated IGCCCG risk group is the calculated risk group based on the present tumor marker levels pre‐chemotherapy. Follow‐up data were available for 224 patients. Patients were treated between 1990 and 2020.

**TABLE 3:** Baseline characteristics of patients in the analysis set.

Included patients	*n* = 523
Age (years) (±SD)	32 (±12)
Clinical stage	*n* (%)
IS	22 (4%)
II	279 (53%)
IIa	144 (28%)
IIb	84 (16%)
IIc	51 (10%)
III	222 (42%)
IIIa	35 (7%)
IIIb	50 (10%)
IIIc	53 (10%)
IIIx	84 (16%)
IGCCCG risk groups	*n* (%)
Good	345 (66%)
Intermediate	116 (22%)
Poor	62 (12%)
Pre‐orchiectomy tumor markers levels	Median [IQR]
AFP (μg/L)	28 (292)
HCG (U/L)	25 (522)
LDH (U/L)	277 (228)
Pre‐chemotherapy tumor markers levels	Median [IQR]
AFP (μg/L)	13 (185)
HCG (U/L)	9 (435)
LDH (U/L)	244 (185)

Abbreviations: AFP, alpha‐fetoprotein; hCG, human chorionic gonadotropin; IGCCCG, International Germ Cell Cancer Collaborative Group; LDH, lactate dehydrogenase; SD, standard deviation.

### 3.2 Changes in IGCCCG risk group using pre‐orchiectomy instead of pre‐chemotherapy marker levels

Using pre‐orchiectomy marker levels would have changed the IGCCCG risk group in 106 (20%) patients. An upgrading (good to intermediate or intermediate to poor prognosis) would have been present in 72 (14%) patients. A downgrading (poor to intermediate or intermediate to good prognosis) would have been present in 34 (7%) patients (Table 4).

**TABLE 4:** Changes in IGCCCG risk group using pre‐orchiectomy instead of pre‐chemotherapy markers.

		Pre‐chemotherapy			
	IGCCCG risk group	Good	Intermediate	Poor	
Pre‐orchiectomy	Good	283	24	3	27 (5.2%)
	Intermediate	58	82	7	7 (1.3%)
	Poor	4	10	52	
		62 (11.9%)	10 (1.9%)		417 (79.7%)
Downstaging with undertreatment		Downstaging with possible undertreatment			
No treatment change					
Upstaging with overtreatment		Upstaging with possible overtreatment			

Abbreviation: IGCCCG, International Germ Cell Cancer Collaborative Group.

### 3.3 Potential therapeutic consequences using pre‐orchiectomy instead of pre‐chemotherapy marker levels

**FIGURE 2:**
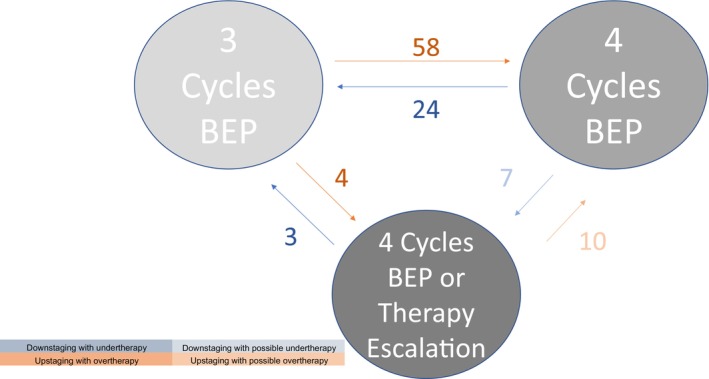
Therapeutic consequences using pre‐orchiectomy instead of pre‐chemotherapy marker levels. BEP, bleomycin, etoposide, and cisplatin. Figure displaying the total number and resulting consequence of using pre‐orchiectomy instead of pre‐chemotherapy markers. Definite overtherapy is displayed in orange, possible overtherapy is showed in pale orange. Definite undertherapy is displayed in blue, possible undertherapy is showed in pale blue. BEP = Bleomycin (B), Etoposid (E) and Cisplatin (P).

Upstaging might have resulted in overtreatment. A change from good to intermediate or poor prognosis would result in 4 instead of 3 cycles of BEP chemotherapy. In our cohort, 62 patients would have received an additional cycle of BEP (Figure 2). A change from intermediate to poor prognosis would only result in a change in therapy in cases where therapy escalation (high‐dose chemotherapy) would be needed (10 cases in our cohort). Therefore, we indicated these cases as “possible overtreatment”.

Accordingly, downstaging might have resulted in undertreatment. Intermediate or poor to good prognosis would have resulted in 3 instead of 4 cycles of BEP. In our cohort, 27 patients would have missed a cycle of BEP (Figure 2). The misclassification of intermediate instead of poor prognosis (7 cases) would have resulted in a change in therapy only in particular cases. Therefore, they are indicated as “possible undertreatment”.

### 3.4 Differences in marker levels pre‐orchiectomy compared to pre‐chemotherapy and influence on classification

Median tumor marker levels were different pre‐orchiectomy compared to pre‐chemotherapy, although this did not result in a change in risk category in most cases: AFP 28 versus 13 μg/L (IQR 186 μg/L), hCG 25 versus 9 U/L (IQR 435 U/L), and LDH 277 versus 244 U/L (IQR 185 U/L) (Table 3). However, for 20 patients, the difference in the AFP levels pre‐orchiectomy compared to pre‐chemotherapy would have resulted in a different IGCCCG classification; similarly, this would apply to 14 patients for hCG levels and 66 patients for LDH levels. The median difference in these 66 patients was 242 U/L (IQR 372 U/L). Only for two of these patients was the difference in LDH levels less than 10%.

**FIGURE 3:**
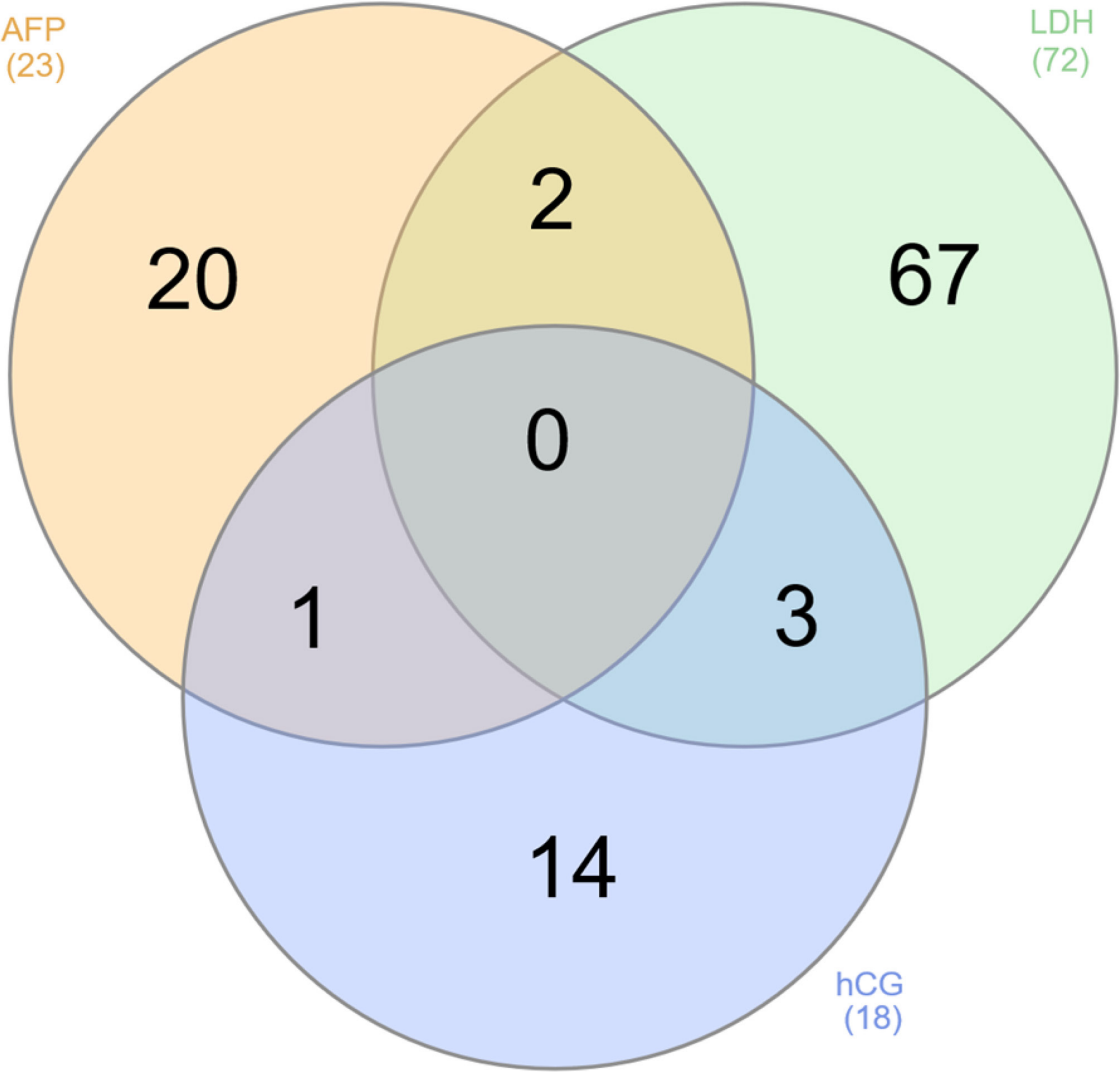
Marker responsible for changes in the IGCCCG risk group. Venn diagram visualizing the causal markers for the change in IGCCCG risk group classification. AFP, alpha‐fetoprotein, hCG, human chorionic gonadotropin, LDH, lactate dehydrogenase.

In six patients, more than one marker level was responsible for a change in classification (Figure 3). Cohen's Kappa between pre‐orchiectomy and pre‐chemotherapy was 0.69 (*p* < 0.001).

### 3.5 Review of correct IGCCCG classification and influence on clinical outcome

Follow‐up data were available for 224 patients. The median follow‐up time was 63 month (IQR 123). Correct IGCCCG classification was reviewed for these patients. If a deviation between the calculated IGCCCG risk group and IGCCCG risk group stated in the clinical data was noted, we double checked for M1b status and extragonadal germ cell tumors as possible explanations. A total of 214 patients showed a correct IGCCCG risk group after review. Ten patients showed a deviation between the stated IGCCCG risk group and the expected IGCCCG risk group based on serum marker levels. For seven patients, nonpulmonary visceral metastases explained the deviation. For the remaining three patients, the deviation was not explainable due to metastases. However, these patients did not present with tumor‐related death in the assessed follow‐up period; therefore, further analysis did not seem reasonable.

## 4 DISCUSSION

Our study confirmed that an incorrect use of pre‐orchiectomy tumor marker levels might result in altered therapy of NSGCT patients because of an improper IGCCCG risk group assignment. We only focused on NSGCT patients, as the tumor marker levels do not impact the prognosis of seminoma patients.^3^, ^14^

To date, only two retrospective single‐center studies have investigated the impact of the misuse of serum tumor marker levels in small cohorts of NSGCT patients (*n* = 83 and *n* = 94).^15^, ^16^ By combining data from eight testis cancer centers, we were able to collect data for a total of 523 evaluable patients with metastasized NSGCT. Clinical data for a complete IGCCCG risk group assignment (location of metastases and site of primary tumor) were only available in one of the previous studies thus far.^16^ In concordance with the former studies, incorrect upstaging by using pre‐orchiectomy tumor marker levels is more frequent (72 patients (14%)) than downstaging (34 patients (7%)), probably because orchiectomy results in a significant tumor mass reduction, followed by a decrease in serum tumor marker levels. For 62 of our patients, incorrect upstaging (intermediate or poor instead of good prognosis) would have resulted in an additional cycle of platinum‐based chemotherapy, usually BEP, deviating from current guideline recommendations.^3^, ^4^ Especially for cisplatin, a cumulative dose effect was observed regarding the risk of secondary malignancies, nephrotoxicity, cardiovascular side effects, paresthesia, or ototoxicity.^7^, ^8^, ^9^, ^11^ Pulmonary toxicity is particularly relevant for bleomycin in a dose‐dependent manner as part of the BEP regimen.^10^

In contrast, 27 of our patients (good instead of intermediate or poor prognosis) would have missed a necessary cycle of platinum‐based chemotherapy.^3^, ^4^ Undertreatment bears the risk of a poorer oncological outcome.

For 224 (43%) patients, follow‐up data were available. Three patients had a deviating IGCCCG risk group not explicable due to distant metastasis other than nonregional lymph nodes and lung or an extragonadal primary tumor. However, none of these three patients presented with a relapse in the follow‐up period. While the explanatory power remains limited, it is well demonstrated that nonguideline concordant therapy results in reduced relapse‐free survival.^17^

The marker most frequently responsible for a deviation between pre‐orchiectomy and pre‐chemotherapy was LDH in 71 cases (66 alone, 5 combined with AFP or hCG). As LDH is an unspecific marker released during tissue damage and particularly hemolysis, it is prone to incorrect measurements. Whether a minimal deviation would lead to a change in therapy is unclear. In our cases, the isolated changes in LDH levels pre‐orchiectomy and pre‐chemotherapy were > 10%, except for two cases, and therefore presumably clinically relevant. The usefulness of LDH, especially for risk stratification, has been reported.^18^

Limitations of this study relate to the retrospective enrollment of patients. Although data from eight testicular cancer centers were combined, it is difficult to draw conclusions, especially regarding the impact on survival due to an altered risk group assignment. An important factor limiting the data quality with respect to long‐term survival outcome may be that follow‐up care is not typically performed at the treating hospital. Additionally, these data were only collected at high‐volume testicular cancer centers with a high awareness of guideline concordant diagnosis and treatment. We hypothesize that the problem of IGCCCG misclassification is more important in hospitals that treat only a few cases of GCT a year, as a discrepancy in the treatment outcome of testicular cancer patients outside of designated centers has typically been reported.^12^ Adding data from medical providers outside of designated testicular cancer centers seems challenging due to the low incidence of testicular cancer in general.

Similarly, different studies have shown that nonguideline concordant treatment of GCT can lead to an impaired clinical outcome. Thibault et al. studied 82 patients who underwent salvage chemotherapy and found that only half of these patients received a guideline‐conforming first‐line treatment.^19^ Similarly, Lin et al. also analyzed 53 relapsed GCT patients and found that 34% of patients had not received appropriate first‐line therapy and were mainly undertreated.^20^ One of the most important reasons for guideline discordance was understaging at diagnosis, resulting in insufficient chemotherapy regimen intensity, which is consistent with our findings of insufficient initial staging. Paffenholz et al. described that nonguideline concordant treatment resulted in a significantly reduced relapse‐free survival.^17^

Due to the involvement of eight different hospitals different assays were used for the serum marker detection. Especially for hCG the use of different assays effects the detection levels,^21^ therefore the combination of different assays has to be considered as limitation. However, guidelines do not take this aspect into consideration.^4^, ^5^

In our study we did not explicitly distinguish between synchronous and metachronous metastases and focused on the clinical stage immediately prior to chemotherapy, but 28 patients presented with a relapse during follow‐up. Predominantly, a synchronous metastatic disease has to be assumed in patients included in our dataset. Future studies might take new serum markers into account.^22^ In particular, microRNA‐371a‐3p has a high potential to alter the daily clinical routine regarding diagnosis and follow‐up. However, microRNA‐371a‐3p has not yet found its way into broad clinical practice due to the lack of prospective clinical trials.

Although a substantial overlap between the pre‐orchiectomy‐ and pre‐chemotherapy‐based IGCCCG risk groups was observed in our data (Cohen's Kappa 0.69; *p* < 0.001), we clearly noted the necessity for a correct IGCCCG classification to avoid nonguideline concordant treatment resulting in supernumerary chemotherapy cycles with the risk of second malignancies and additional adverse side effects or an undertreatment with a worse oncological outcome.

Taken together, using tumor marker levels at the time of orchiectomy bears a relevant risk of inappropriate IGCCCG classification, resulting in potentially impaired therapy. Therefore, it is important to further deliver the basics of testicular cancer guidelines into primary care and/or to strengthen efforts for the centralization of therapy.

## AUTHOR CONTRIBUTIONS

**Matthäeus Majewski:** Conceptualization (equal); data curation (equal); formal analysis (equal); visualization (equal); writing – original draft (equal). **Pia Paffenholz:** Data curation (equal); writing – review and editing (equal). **Christian Ruf:** Writing – review and editing (equal). **Yue Che:** Data curation (equal); writing – review and editing (equal). **Christoph Seidel:** Data curation (equal); writing – review and editing (equal). **Julia Heinzelbecker:** Data curation (equal); writing – review and editing (equal). **Hans‐Ulrich Schmelz:** Writing – review and editing (equal). **Cord Matthies:** Data curation (equal); writing – review and editing (equal). **Peter Albers:** Writing – review and editing (equal). **Carsten Bokemeyer:** Data curation (equal); writing – review and editing (equal). **Axel Heidenreich:** Writing – review and editing (equal). **Martin Pichler:** Data curation (equal); writing – review and editing (equal). **Tim Nestler:** Conceptualization (equal); data curation (equal); formal analysis (equal); supervision (equal); writing – original draft (equal); writing – review and editing (equal).

## CONFLICT OF INTEREST STATEMENT

The authors have no conflicts of interest to declare.

## TAKE HOME MESSAGE

Using pre‐orchiectomy tumor marker levels for IGCCCG risk group assignment might result in nonguideline concordant treatment. In most cases, this results in overtreatment.

## Data Availability

The data that support the findings of this study are available on request from the corresponding author. The data are not publicly available due to privacy or ethical restrictions.
